# 9-[3-(Carbazol-9-yl)-5-methyl­phen­yl]carbazole

**DOI:** 10.1107/S1600536813011641

**Published:** 2013-05-04

**Authors:** Jae Eun Kim, Jun Hee Kim, Wonbo Sim, Jai Young Lee

**Affiliations:** a Department of Chemistry, Konyang University, Nonsan 320-711, Republic of Korea

## Abstract

The title compound, C_31_H_22_N_2_, crystallizes with two symmetry-independent mol­ecules in the asymmetric unit. The mol­ecules have slightly different conformations, the dihedral angles between the central phenyl ring and the carbazolyl groups being 56.29 (4) and 59.57 (4)° in one mol­ecule and 48.71 (4) and 65.47 (4)° in the other. In the crystal, mol­ecules are linked by weak C—H⋯π and π–π [centroid–centroid distances = 3.7698 (10), 3.8292 (9), 3.9429 (10) and 3.9431 (10) Å].

## Related literature

For the preparation of the title compound, see: Kwon *et al.* (2007 ▶). For the structure of the related compound 1,3-bis­(carbazol-9-yl)benzene, see: Sun *et al.* (2006 ▶).
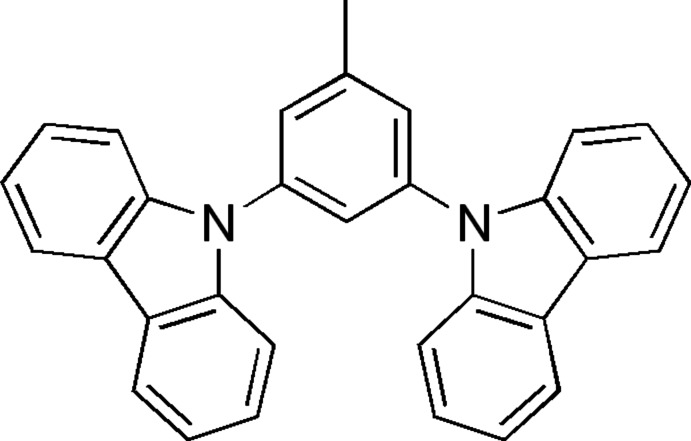


## Experimental

### 

#### Crystal data

C_31_H_22_N_2_
*M* *_r_* = 422.51
Monoclinic, 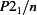

*a* = 9.2503 (1) Å
*b* = 29.3854 (4) Å
*c* = 16.2616 (2) Å
β = 96.901 (1)°
*V* = 4388.27 (9) Å^3^
*Z* = 8
Mo *K*α radiation
μ = 0.08 mm^−1^
*T* = 173 K
0.26 × 0.23 × 0.22 mm

#### Data collection

Bruker APEXII CCD diffractometer
Absorption correction: multi-scan (*SADABS*; Sheldrick, 1996 ▶) *T*_min_ = 0.981, *T*_max_ = 0.984
42985 measured reflections
10834 independent reflections
7284 reflections with *I* > 2σ(*I*)
*R*_int_ = 0.041

#### Refinement

*R*[*F* ^2^ > 2σ(*F* ^2^)] = 0.051
*wR*(*F* ^2^) = 0.117
*S* = 1.03
10834 reflections
598 parameters
H-atom parameters constrained
Δρ_max_ = 0.26 e Å^−3^
Δρ_min_ = −0.22 e Å^−3^

### 

Data collection: *APEX2* (Bruker, 2006 ▶); cell refinement: *SAINT-Plus* (Bruker, 2006 ▶); data reduction: *SAINT-Plus*; program(s) used to solve structure: *SHELXTL* (Sheldrick, 2008 ▶); program(s) used to refine structure: *SHELXTL*; molecular graphics: *SHELXTL*; software used to prepare material for publication: *SHELXTL*.

## Supplementary Material

Crystal structure: contains datablock(s) I, global. DOI: 10.1107/S1600536813011641/fb2283sup1.cif

Structure factors: contains datablock(s) I. DOI: 10.1107/S1600536813011641/fb2283Isup2.hkl

Supplementary material file. DOI: 10.1107/S1600536813011641/fb2283Isup3.cml

Additional supplementary materials:  crystallographic information; 3D view; checkCIF report

## Figures and Tables

**Table 1:** C—H⋯π (Å, °) *Cg*1, *Cg*3—*Cg*6 and *Cg*9 are the centroids of the C44–C49, C7–C12, C56–C61, C1–C6, C19–C24 and C32–C37 benzene rings while *Cg*2, *Cg*7 and *Cg*8 are the centroids of the N4/C50/C55/C56/C61, N2/C19/C24/C25/C30 and N3/C32/C37/C38/C43 pyrrole rings.

C—H⋯π	*C*—H	H⋯*Cg*	*C*⋯*Cg*	*C*—H⋯*Cg*
C9—H9*A*⋯*Cg*1^i^	0.95	2.96	3.776 (2)	145
C5—H5*A*⋯*Cg*2^ii^	0.95	2.77	3.6969 (19)	165
C53—H53*A*⋯*Cg*3^iii^	0.95	2.76	3.5983 (19)	148
C8—H8*A*⋯*Cg*4^ii^	0.95	2.81	3.7122 (19)	160
C49—H49*A*⋯*Cg*5	0.95	2.98	3.8654 (16)	156
C57—H57*A*⋯*Cg*6^iv^	0.95	2.85	3.3776 (17)	117
C58—H58*A*⋯*Cg*7^iv^	0.95	2.88	3.3749 (18)	114
C59—H59*A*⋯*Cg*8^v^	0.95	2.66	3.5084 (18)	149
C60—H60*A*⋯*Cg*9^v^	0.95	2.74	3.5180 (17)	140

Symmetry codes: (i) 
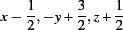
; (ii) 
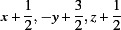
; (iii) 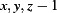; (iv) 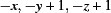; (v) 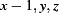.

**Fig. 1.**
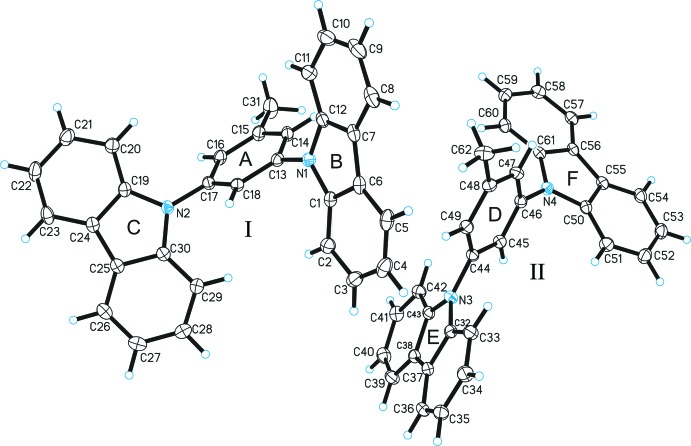
The title molecules with the atom-numbering scheme and assigned labels to the rings (see text). The displacement ellipsoids are drawn at the 50% probability level. The H atoms are presented as small spheres of arbitrary radius.

**Fig. 2.**
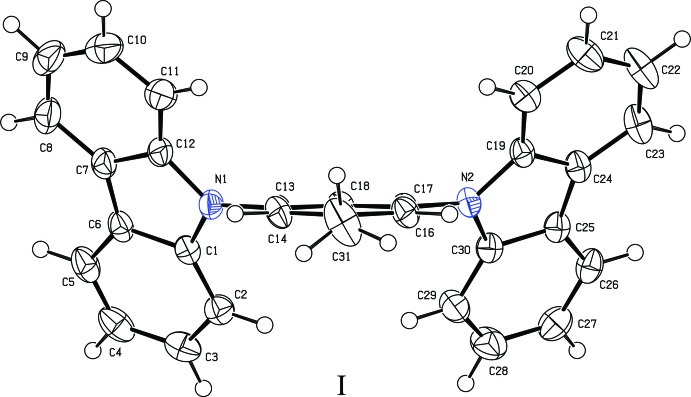
The first conformer. The displacement ellipsoids are drawn at the 50% probability level. The H atoms are presented as small spheres of arbitrary radius.

**Fig. 3.**
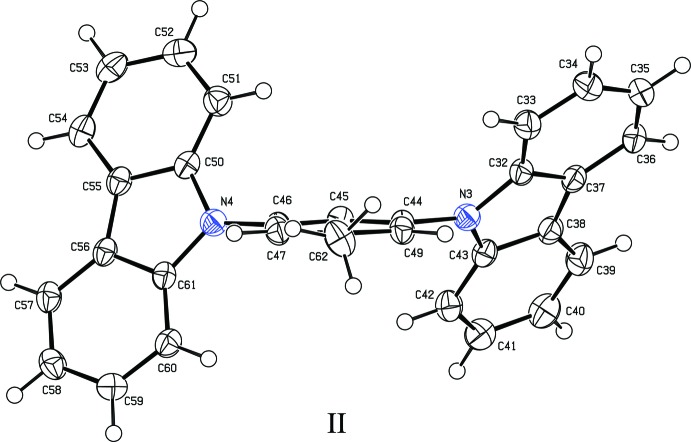
The second conformer. The displacement ellipsoids are drawn at the 50% probability level. The H atoms are presented as small spheres of arbitrary radius.

**Fig. 4.**
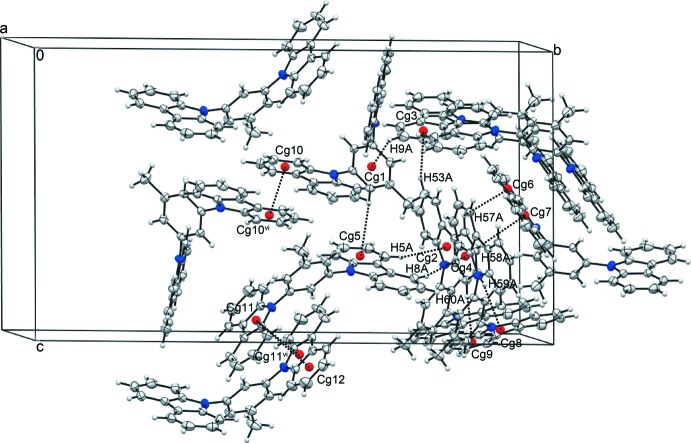
A view of the C—H···π interactions and π-electron ··· π-electron ring interactions (dotted lines) in the crystal structure of the title compound. Symmetry codes: (i) x - 1/2, -y + 3/2, z + 1/2; (ii) x + 1/2, -y + 3/2, z + 1/2; (iii) x, y, z - 1; (iv) -x, -y + 1, -z + 1; (v) x - 1, y, z; (vi) -x + 1, -y + 1, -z + 1; (vii) x + 3/2, -y + 3/2, z + 5/2.

## supplementary crystallographic information

### Comment

The title compound, 5-*m*CP (systematic name: 5-methyl-*N*,*N*-dicarbazolyl-1,3-benzene), had been shown to be better for fluorescent blue light-emitting dopants (Kwon *et al.*, 2007). However, its crystal structure has not been reported yet, which motivated this study.

The title structure (Fig. 1) is composed of two symmetry independent molecules each of which has a different conformation. The conformers can be compared by Figs. 2 and 3. Specifically, the difference between the conformers is manifested by interplanar angles between the central phenyl fragment and the carbazolyl groups. These angles (see Fig. 1) are equal to 56.29 (4) ° (A—B), 59.57 (4) ° (A—C), 48.71 (4) ° (D—E) and 65.47 (4)° (D—F). All the bond lengths and the bond angles are normal and comparable to those observed in a similar structure of *m*-CP = 1,3-bis(carbazol-9-yl)benzene (Sun *et al.*, 2006).

In the crystal structure, the molecules are linked by weak intermolecular C—H···π-electron ring interactions - see Fig. 4 and Table 1. In addition, there are also present π-electron···π-electron ring interactions in the structure. Their overview is given in Table 2 and also shown in Fig. 4.

### Experimental

The title compound was prepared by the reaction of carbazole with 1,3-dibromo-5-methylbenzene in toluene according to a method reported by Kwon *et al.* (2007). Single crystals of the title compound for X-ray analysis were obtained by slow evaporation of the CH_2_Cl_2_ solution. The single crystals were block-like (0.4×0.2×0.2 mm) and colourless.

### Refinement

All H atoms were discernible in the difference electron-density map. However, they were situated in idealized positions and refined with the following constraints: C—H = 0.95 Å and *U*_iso_(H) = 1.2*U*_eq_(C) for aryl H atoms, and C—H = 0.98 Å and *U*_iso_(H) = 1.5*U*_eq_(C) for methyl H atoms. The methyl groups were allowed to rotate about the C—C_methyl_ bonds using the function AFIX 137 of *SHELXL97* (Sheldrick, 2008).

### Crystal data

**Table:**

C_31_H_22_N_2_	*F*(000) = 1776
*M**_r_* = 422.51	*D*_x_ = 1.279 Mg m^−^^3^
Monoclinic, *P*2_1_/*n*	Mo *K*α radiation, λ = 0.71073 Å
Hall symbol: -P 2yn	Cell parameters from 8960 reflections
*a* = 9.2503 (1) Å	θ = 2.3–25.3°
*b* = 29.3854 (4) Å	µ = 0.08 mm^−^^1^
*c* = 16.2616 (2) Å	*T* = 173 K
β = 96.901 (1)°	Block, colourless
*V* = 4388.27 (9) Å^3^	0.26 × 0.23 × 0.22 mm
*Z* = 8	

### Data collection

**Table:**

Bruker APEXII CCD diffractometer	10834 independent reflections
Radiation source: fine-focus sealed tube	7284 reflections with *I* > 2σ(*I*)
Graphite monochromator	*R*_int_ = 0.041
φ and ω scans	θ_max_ = 28.3°, θ_min_ = 1.4°
Absorption correction: multi-scan (*SADABS*; Sheldrick, 1996)	*h* = −11→12
*T*_min_ = 0.981, *T*_max_ = 0.984	*k* = −39→33
42985 measured reflections	*l* = −21→21

### Refinement

**Table:**

Refinement on *F*^2^	Secondary atom site location: difference Fourier map
Least-squares matrix: full	Hydrogen site location: difference Fourier map
*R*[*F*^2^ > 2σ(*F*^2^)] = 0.051	H-atom parameters constrained
*wR*(*F*^2^) = 0.117	*w* = 1/[σ^2^(*F*_o_^2^) + (0.0411*P*)^2^ + 1.3445*P*] where *P* = (*F*_o_^2^ + 2*F*_c_^2^)/3
*S* = 1.03	(Δ/σ)_max_ = 0.001
10834 reflections	Δρ_max_ = 0.26 e Å^−^^3^
598 parameters	Δρ_min_ = −0.22 e Å^−^^3^
0 restraints	Extinction correction: *SHELXTL* (Sheldrick, 2008), Fc^*^=kFc[1+0.001xFc^2^λ^3^/sin(2θ)]^-1/4^
174 constraints	Extinction coefficient: 0.0025 (3)
Primary atom site location: structure-invariant direct methods	

### Special details

**Table:**

Geometry. All e.s.d.'s (except the e.s.d. in the dihedral angle between two l.s. planes) are estimated using the full covariance matrix. The cell e.s.d.'s are taken into account individually in the estimation of e.s.d.'s in distances, angles and torsion angles; correlations between e.s.d.'s in cell parameters are only used when they are defined by crystal symmetry. An approximate (isotropic) treatment of cell e.s.d.'s is used for estimating e.s.d.'s involving l.s. planes.
Refinement. Refinement of *F*^2^ against ALL reflections. The weighted *R*-factor *wR* and goodness of fit *S* are based on *F*^2^, conventional *R*-factors *R* are based on *F*, with *F* set to zero for negative *F*^2^. The threshold expression of *F*^2^ > σ(*F*^2^) is used only for calculating *R*-factors(gt) *etc*. and is not relevant to the choice of reflections for refinement. *R*-factors based on *F*^2^ are statistically about twice as large as those based on *F*, and *R*- factors based on ALL data will be even larger.

### Fractional atomic coordinates and isotropic or equivalent isotropic displacement parameters (Å^2^)

**Table:**

	*x*	*y*	*z*	*U*_iso_*/*U*_eq_	
N1	0.27690 (14)	0.62649 (4)	0.76286 (8)	0.0290 (3)	
N2	0.33297 (14)	0.47722 (4)	0.89106 (8)	0.0275 (3)	
N3	0.57996 (13)	0.61483 (4)	0.44472 (8)	0.0274 (3)	
N4	0.16834 (14)	0.66661 (4)	0.25357 (8)	0.0279 (3)	
C1	0.41194 (17)	0.64001 (5)	0.74124 (9)	0.0271 (4)	
C2	0.52539 (18)	0.61396 (6)	0.71889 (10)	0.0329 (4)	
H2A	0.5201	0.5817	0.7181	0.039*	
C3	0.64689 (19)	0.63649 (7)	0.69772 (10)	0.0385 (4)	
H3A	0.7256	0.6193	0.6814	0.046*	
C4	0.6567 (2)	0.68386 (7)	0.69976 (11)	0.0421 (5)	
H4A	0.7417	0.6984	0.6852	0.051*	
C5	0.5443 (2)	0.70945 (6)	0.72263 (10)	0.0384 (4)	
H5A	0.5512	0.7417	0.7241	0.046*	
C6	0.41973 (18)	0.68787 (5)	0.74376 (9)	0.0306 (4)	
C7	0.28475 (19)	0.70389 (6)	0.76935 (10)	0.0322 (4)	
C8	0.2317 (2)	0.74691 (6)	0.78619 (11)	0.0431 (5)	
H8A	0.2878	0.7734	0.7793	0.052*	
C9	0.0967 (2)	0.75024 (7)	0.81284 (11)	0.0493 (5)	
H9A	0.0606	0.7793	0.8258	0.059*	
C10	0.0120 (2)	0.71195 (7)	0.82116 (11)	0.0468 (5)	
H10A	−0.0820	0.7154	0.8382	0.056*	
C11	0.06163 (19)	0.66884 (6)	0.80515 (11)	0.0385 (4)	
H11A	0.0038	0.6426	0.8114	0.046*	
C12	0.19901 (18)	0.66530 (5)	0.77966 (10)	0.0306 (4)	
C13	0.22740 (16)	0.58089 (5)	0.77080 (9)	0.0267 (3)	
C14	0.09856 (17)	0.56694 (6)	0.72562 (10)	0.0315 (4)	
H14A	0.0448	0.5877	0.6890	0.038*	
C15	0.04736 (17)	0.52304 (6)	0.73321 (10)	0.0334 (4)	
C16	0.12611 (17)	0.49316 (6)	0.78761 (10)	0.0309 (4)	
H16A	0.0922	0.4629	0.7933	0.037*	
C17	0.25452 (16)	0.50739 (5)	0.83373 (9)	0.0267 (3)	
C18	0.30585 (16)	0.55118 (5)	0.82526 (9)	0.0258 (3)	
H18A	0.3940	0.5608	0.8565	0.031*	
C19	0.27761 (17)	0.45455 (5)	0.95570 (9)	0.0267 (4)	
C20	0.14002 (18)	0.45643 (6)	0.98060 (10)	0.0341 (4)	
H20A	0.0660	0.4751	0.9526	0.041*	
C21	0.1141 (2)	0.43014 (7)	1.04771 (11)	0.0434 (5)	
H21A	0.0208	0.4310	1.0665	0.052*	
C22	0.2218 (2)	0.40240 (7)	1.08835 (11)	0.0460 (5)	
H22A	0.2004	0.3844	1.1338	0.055*	
C23	0.35877 (19)	0.40069 (6)	1.06370 (10)	0.0373 (4)	
H23A	0.4317	0.3816	1.0917	0.045*	
C24	0.38880 (17)	0.42728 (5)	0.99720 (9)	0.0283 (4)	
C25	0.51718 (17)	0.43418 (5)	0.95646 (10)	0.0275 (4)	
C26	0.65750 (18)	0.41636 (6)	0.96810 (11)	0.0337 (4)	
H26A	0.6850	0.3955	1.0118	0.040*	
C27	0.75564 (19)	0.42934 (6)	0.91567 (12)	0.0404 (4)	
H27A	0.8517	0.4173	0.9232	0.048*	
C28	0.7160 (2)	0.45993 (7)	0.85166 (12)	0.0464 (5)	
H28A	0.7860	0.4685	0.8163	0.056*	
C29	0.57758 (19)	0.47825 (6)	0.83807 (11)	0.0391 (4)	
H29A	0.5511	0.4989	0.7939	0.047*	
C30	0.47897 (17)	0.46520 (5)	0.89147 (10)	0.0285 (4)	
C31	−0.0906 (2)	0.50771 (8)	0.68137 (13)	0.0543 (6)	
H31A	−0.0960	0.5216	0.6263	0.081*	
H31B	−0.1752	0.5171	0.7082	0.081*	
H31C	−0.0901	0.4745	0.6761	0.081*	
C32	0.71967 (16)	0.62901 (5)	0.47688 (9)	0.0268 (3)	
C33	0.77012 (18)	0.67215 (6)	0.49994 (10)	0.0318 (4)	
H33A	0.7071	0.6978	0.4953	0.038*	
C34	0.91550 (18)	0.67640 (6)	0.52998 (11)	0.0368 (4)	
H34A	0.9522	0.7055	0.5471	0.044*	
C35	1.01002 (18)	0.63939 (6)	0.53595 (11)	0.0378 (4)	
H35A	1.1094	0.6435	0.5573	0.045*	
C36	0.95993 (17)	0.59695 (6)	0.51104 (10)	0.0341 (4)	
H36A	1.0247	0.5717	0.5141	0.041*	
C37	0.81306 (17)	0.59115 (5)	0.48111 (9)	0.0283 (4)	
C38	0.72705 (17)	0.55249 (6)	0.45035 (9)	0.0286 (4)	
C39	0.75808 (19)	0.50671 (6)	0.44074 (11)	0.0362 (4)	
H39A	0.8549	0.4958	0.4534	0.043*	
C40	0.6475 (2)	0.47731 (6)	0.41274 (11)	0.0379 (4)	
H40A	0.6679	0.4459	0.4060	0.045*	
C41	0.50542 (19)	0.49329 (6)	0.39419 (10)	0.0351 (4)	
H41A	0.4300	0.4723	0.3761	0.042*	
C42	0.47147 (18)	0.53863 (6)	0.40137 (10)	0.0318 (4)	
H42A	0.3746	0.5493	0.3879	0.038*	
C43	0.58400 (17)	0.56821 (5)	0.42906 (9)	0.0268 (3)	
C44	0.45648 (16)	0.64348 (5)	0.42645 (9)	0.0251 (3)	
C45	0.37372 (16)	0.64064 (5)	0.34928 (9)	0.0268 (3)	
H45A	0.3985	0.6195	0.3090	0.032*	
C46	0.25485 (16)	0.66907 (5)	0.33223 (9)	0.0259 (3)	
C47	0.22033 (17)	0.70061 (5)	0.39003 (10)	0.0282 (4)	
H47A	0.1394	0.7203	0.3769	0.034*	
C48	0.30322 (16)	0.70377 (5)	0.46735 (10)	0.0278 (4)	
C49	0.41995 (16)	0.67422 (5)	0.48516 (9)	0.0276 (4)	
H49A	0.4754	0.6751	0.5382	0.033*	
C50	0.21311 (17)	0.67856 (5)	0.17760 (9)	0.0264 (3)	
C51	0.34967 (18)	0.69130 (6)	0.15945 (10)	0.0334 (4)	
H51A	0.4302	0.6931	0.2015	0.040*	
C52	0.3645 (2)	0.70125 (6)	0.07790 (11)	0.0378 (4)	
H52A	0.4574	0.7097	0.0635	0.045*	
C53	0.2460 (2)	0.69919 (6)	0.01607 (11)	0.0378 (4)	
H53A	0.2591	0.7067	−0.0394	0.045*	
C54	0.11100 (19)	0.68644 (6)	0.03460 (10)	0.0331 (4)	
H54A	0.0309	0.6849	−0.0078	0.040*	
C55	0.09246 (17)	0.67578 (5)	0.11612 (9)	0.0271 (4)	
C56	−0.02945 (17)	0.66053 (5)	0.15654 (9)	0.0251 (3)	
C57	−0.17423 (17)	0.64988 (5)	0.12858 (10)	0.0303 (4)	
H57A	−0.2104	0.6531	0.0717	0.036*	
C58	−0.26369 (18)	0.63463 (6)	0.18442 (10)	0.0328 (4)	
H58A	−0.3625	0.6276	0.1659	0.039*	
C59	−0.21114 (18)	0.62944 (6)	0.26783 (10)	0.0320 (4)	
H59A	−0.2747	0.6185	0.3051	0.038*	
C60	−0.06856 (17)	0.63980 (5)	0.29759 (10)	0.0296 (4)	
H60A	−0.0333	0.6364	0.3546	0.036*	
C61	0.02111 (16)	0.65531 (5)	0.24095 (9)	0.0252 (3)	
C62	0.26782 (18)	0.73838 (6)	0.53012 (11)	0.0387 (4)	
H62A	0.3574	0.7538	0.5535	0.058*	
H62B	0.2237	0.7231	0.5745	0.058*	
H62C	0.1994	0.7608	0.5032	0.058*	

### Atomic displacement parameters (Å^2^)

**Table:**

	*U*^11^	*U*^22^	*U*^33^	*U*^12^	*U*^13^	*U*^23^
N1	0.0300 (7)	0.0231 (7)	0.0347 (8)	−0.0001 (6)	0.0066 (6)	0.0044 (6)
N2	0.0270 (7)	0.0256 (7)	0.0297 (7)	−0.0013 (6)	0.0029 (5)	0.0063 (6)
N3	0.0253 (7)	0.0278 (7)	0.0281 (7)	0.0024 (6)	−0.0006 (5)	−0.0001 (6)
N4	0.0260 (7)	0.0341 (8)	0.0226 (7)	−0.0002 (6)	−0.0019 (5)	0.0016 (6)
C1	0.0302 (8)	0.0262 (9)	0.0244 (8)	−0.0037 (7)	0.0012 (6)	0.0026 (6)
C2	0.0336 (9)	0.0333 (10)	0.0314 (9)	−0.0012 (7)	0.0027 (7)	0.0000 (7)
C3	0.0318 (9)	0.0527 (12)	0.0314 (9)	−0.0029 (8)	0.0053 (7)	0.0001 (8)
C4	0.0402 (11)	0.0546 (13)	0.0311 (9)	−0.0194 (9)	0.0026 (8)	0.0039 (9)
C5	0.0513 (11)	0.0323 (10)	0.0304 (9)	−0.0164 (9)	0.0001 (8)	0.0034 (7)
C6	0.0412 (10)	0.0268 (9)	0.0224 (8)	−0.0057 (7)	−0.0015 (7)	0.0034 (7)
C7	0.0435 (10)	0.0273 (9)	0.0245 (8)	0.0028 (8)	−0.0009 (7)	0.0045 (7)
C8	0.0641 (13)	0.0278 (10)	0.0349 (10)	0.0074 (9)	−0.0047 (9)	0.0029 (8)
C9	0.0687 (14)	0.0404 (12)	0.0359 (10)	0.0258 (11)	−0.0058 (10)	−0.0023 (9)
C10	0.0467 (11)	0.0603 (14)	0.0322 (10)	0.0242 (10)	−0.0006 (8)	−0.0010 (9)
C11	0.0357 (10)	0.0443 (11)	0.0356 (10)	0.0075 (8)	0.0045 (8)	0.0041 (8)
C12	0.0377 (9)	0.0279 (9)	0.0253 (8)	0.0069 (7)	0.0008 (7)	0.0048 (7)
C13	0.0274 (8)	0.0253 (8)	0.0279 (8)	−0.0028 (6)	0.0057 (6)	0.0018 (7)
C14	0.0299 (9)	0.0352 (10)	0.0286 (8)	−0.0011 (7)	0.0006 (7)	0.0104 (7)
C15	0.0284 (9)	0.0415 (10)	0.0294 (9)	−0.0083 (7)	−0.0003 (7)	0.0059 (7)
C16	0.0319 (9)	0.0298 (9)	0.0310 (9)	−0.0087 (7)	0.0036 (7)	0.0032 (7)
C17	0.0273 (8)	0.0267 (9)	0.0258 (8)	0.0000 (7)	0.0019 (6)	0.0024 (6)
C18	0.0238 (8)	0.0271 (9)	0.0261 (8)	−0.0031 (6)	0.0013 (6)	0.0005 (7)
C19	0.0295 (8)	0.0227 (8)	0.0274 (8)	−0.0066 (6)	0.0008 (6)	0.0014 (6)
C20	0.0311 (9)	0.0378 (10)	0.0330 (9)	−0.0050 (7)	0.0017 (7)	0.0021 (8)
C21	0.0337 (10)	0.0593 (13)	0.0376 (10)	−0.0125 (9)	0.0054 (8)	0.0075 (9)
C22	0.0421 (11)	0.0604 (13)	0.0337 (10)	−0.0196 (9)	−0.0027 (8)	0.0184 (9)
C23	0.0363 (10)	0.0376 (10)	0.0346 (9)	−0.0121 (8)	−0.0096 (8)	0.0103 (8)
C24	0.0304 (8)	0.0247 (9)	0.0283 (8)	−0.0079 (7)	−0.0032 (7)	0.0011 (7)
C25	0.0279 (8)	0.0236 (8)	0.0294 (8)	−0.0042 (7)	−0.0026 (7)	−0.0011 (7)
C26	0.0350 (9)	0.0256 (9)	0.0380 (9)	−0.0003 (7)	−0.0058 (8)	−0.0006 (7)
C27	0.0313 (9)	0.0391 (11)	0.0499 (11)	0.0053 (8)	0.0009 (8)	−0.0043 (9)
C28	0.0362 (10)	0.0573 (13)	0.0480 (11)	0.0026 (9)	0.0148 (9)	0.0081 (10)
C29	0.0369 (10)	0.0436 (11)	0.0376 (10)	0.0027 (8)	0.0083 (8)	0.0111 (8)
C30	0.0279 (8)	0.0264 (9)	0.0304 (8)	−0.0023 (7)	0.0002 (7)	0.0004 (7)
C31	0.0431 (11)	0.0686 (14)	0.0465 (11)	−0.0238 (10)	−0.0141 (9)	0.0196 (10)
C32	0.0250 (8)	0.0328 (9)	0.0220 (8)	0.0004 (7)	0.0005 (6)	0.0030 (7)
C33	0.0295 (9)	0.0317 (9)	0.0337 (9)	0.0019 (7)	0.0018 (7)	0.0015 (7)
C34	0.0322 (9)	0.0358 (10)	0.0414 (10)	−0.0052 (8)	0.0003 (8)	0.0024 (8)
C35	0.0259 (9)	0.0459 (11)	0.0397 (10)	−0.0045 (8)	−0.0031 (7)	0.0110 (8)
C36	0.0279 (9)	0.0378 (10)	0.0355 (9)	0.0058 (7)	−0.0005 (7)	0.0120 (8)
C37	0.0292 (8)	0.0320 (9)	0.0236 (8)	0.0033 (7)	0.0023 (6)	0.0064 (7)
C38	0.0301 (9)	0.0316 (9)	0.0239 (8)	0.0029 (7)	0.0024 (6)	0.0042 (7)
C39	0.0347 (9)	0.0350 (10)	0.0383 (10)	0.0081 (8)	0.0021 (8)	0.0035 (8)
C40	0.0491 (11)	0.0285 (10)	0.0359 (10)	0.0065 (8)	0.0043 (8)	−0.0017 (8)
C41	0.0411 (10)	0.0327 (10)	0.0308 (9)	−0.0033 (8)	0.0010 (7)	−0.0039 (7)
C42	0.0313 (9)	0.0348 (10)	0.0288 (8)	0.0021 (7)	0.0006 (7)	−0.0023 (7)
C43	0.0306 (8)	0.0287 (9)	0.0208 (7)	0.0024 (7)	0.0022 (6)	0.0009 (6)
C44	0.0223 (8)	0.0262 (8)	0.0263 (8)	0.0006 (6)	0.0003 (6)	0.0027 (6)
C45	0.0293 (8)	0.0261 (9)	0.0247 (8)	0.0008 (7)	0.0025 (6)	−0.0021 (6)
C46	0.0244 (8)	0.0282 (9)	0.0241 (8)	−0.0019 (6)	−0.0012 (6)	0.0020 (6)
C47	0.0232 (8)	0.0285 (9)	0.0321 (9)	0.0025 (7)	0.0002 (6)	0.0000 (7)
C48	0.0249 (8)	0.0291 (9)	0.0295 (8)	−0.0023 (7)	0.0033 (6)	−0.0028 (7)
C49	0.0257 (8)	0.0322 (9)	0.0240 (8)	−0.0018 (7)	−0.0009 (6)	−0.0005 (7)
C50	0.0317 (9)	0.0241 (8)	0.0230 (8)	0.0017 (7)	0.0014 (6)	0.0008 (6)
C51	0.0323 (9)	0.0338 (10)	0.0334 (9)	−0.0012 (7)	0.0009 (7)	−0.0008 (7)
C52	0.0396 (10)	0.0344 (10)	0.0407 (10)	−0.0033 (8)	0.0106 (8)	0.0033 (8)
C53	0.0478 (11)	0.0384 (10)	0.0283 (9)	0.0031 (8)	0.0085 (8)	0.0066 (8)
C54	0.0385 (10)	0.0347 (10)	0.0250 (8)	0.0046 (8)	−0.0003 (7)	0.0029 (7)
C55	0.0316 (9)	0.0228 (8)	0.0262 (8)	0.0033 (7)	0.0006 (7)	0.0000 (6)
C56	0.0302 (8)	0.0198 (8)	0.0242 (8)	0.0033 (6)	−0.0008 (6)	−0.0003 (6)
C57	0.0324 (9)	0.0279 (9)	0.0285 (8)	0.0017 (7)	−0.0049 (7)	−0.0016 (7)
C58	0.0279 (8)	0.0318 (9)	0.0369 (9)	−0.0023 (7)	−0.0027 (7)	−0.0047 (7)
C59	0.0330 (9)	0.0314 (9)	0.0322 (9)	−0.0032 (7)	0.0067 (7)	−0.0001 (7)
C60	0.0338 (9)	0.0297 (9)	0.0246 (8)	0.0003 (7)	0.0005 (7)	0.0018 (7)
C61	0.0266 (8)	0.0229 (8)	0.0249 (8)	0.0025 (6)	−0.0012 (6)	−0.0003 (6)
C62	0.0311 (9)	0.0440 (11)	0.0402 (10)	0.0024 (8)	0.0007 (8)	−0.0143 (8)

### Geometric parameters (Å, º)

**Table:**

N1—C12	1.393 (2)	C28—H28A	0.9500
N1—C1	1.396 (2)	C29—C30	1.387 (2)
N1—C13	1.427 (2)	C29—H29A	0.9500
N2—C19	1.393 (2)	C31—H31A	0.9800
N2—C30	1.395 (2)	C31—H31B	0.9800
N2—C17	1.4210 (19)	C31—H31C	0.9800
N3—C43	1.395 (2)	C32—C33	1.387 (2)
N3—C32	1.3981 (19)	C32—C37	1.405 (2)
N3—C44	1.4213 (19)	C33—C34	1.380 (2)
N4—C61	1.3928 (19)	C33—H33A	0.9500
N4—C50	1.394 (2)	C34—C35	1.392 (2)
N4—C46	1.4269 (18)	C34—H34A	0.9500
C1—C2	1.382 (2)	C35—C36	1.374 (2)
C1—C6	1.409 (2)	C35—H35A	0.9500
C2—C3	1.383 (2)	C36—C37	1.397 (2)
C2—H2A	0.9500	C36—H36A	0.9500
C3—C4	1.395 (3)	C37—C38	1.442 (2)
C3—H3A	0.9500	C38—C39	1.388 (2)
C4—C5	1.370 (3)	C38—C43	1.405 (2)
C4—H4A	0.9500	C39—C40	1.374 (2)
C5—C6	1.394 (2)	C39—H39A	0.9500
C5—H5A	0.9500	C40—C41	1.394 (2)
C6—C7	1.442 (2)	C40—H40A	0.9500
C7—C8	1.395 (2)	C41—C42	1.377 (2)
C7—C12	1.405 (2)	C41—H41A	0.9500
C8—C9	1.374 (3)	C42—C43	1.389 (2)
C8—H8A	0.9500	C42—H42A	0.9500
C9—C10	1.387 (3)	C44—C49	1.385 (2)
C9—H9A	0.9500	C44—C45	1.392 (2)
C10—C11	1.383 (3)	C45—C46	1.382 (2)
C10—H10A	0.9500	C45—H45A	0.9500
C11—C12	1.387 (2)	C46—C47	1.384 (2)
C11—H11A	0.9500	C47—C48	1.395 (2)
C13—C14	1.385 (2)	C47—H47A	0.9500
C13—C18	1.385 (2)	C48—C49	1.389 (2)
C14—C15	1.385 (2)	C48—C62	1.505 (2)
C14—H14A	0.9500	C49—H49A	0.9500
C15—C16	1.389 (2)	C50—C51	1.383 (2)
C15—C31	1.511 (2)	C50—C55	1.408 (2)
C16—C17	1.391 (2)	C51—C52	1.381 (2)
C16—H16A	0.9500	C51—H51A	0.9500
C17—C18	1.384 (2)	C52—C53	1.397 (2)
C18—H18A	0.9500	C52—H52A	0.9500
C19—C20	1.382 (2)	C53—C54	1.372 (2)
C19—C24	1.410 (2)	C53—H53A	0.9500
C20—C21	1.381 (2)	C54—C55	1.393 (2)
C20—H20A	0.9500	C54—H54A	0.9500
C21—C22	1.391 (3)	C55—C56	1.443 (2)
C21—H21A	0.9500	C56—C57	1.397 (2)
C22—C23	1.375 (3)	C56—C61	1.404 (2)
C22—H22A	0.9500	C57—C58	1.375 (2)
C23—C24	1.389 (2)	C57—H57A	0.9500
C23—H23A	0.9500	C58—C59	1.393 (2)
C24—C25	1.442 (2)	C58—H58A	0.9500
C25—C26	1.391 (2)	C59—C60	1.383 (2)
C25—C30	1.408 (2)	C59—H59A	0.9500
C26—C27	1.372 (3)	C60—C61	1.388 (2)
C26—H26A	0.9500	C60—H60A	0.9500
C27—C28	1.391 (3)	C62—H62A	0.9800
C27—H27A	0.9500	C62—H62B	0.9800
C28—C29	1.382 (2)	C62—H62C	0.9800
			
C12—N1—C1	108.42 (13)	C15—C31—H31A	109.5
C12—N1—C13	124.87 (14)	C15—C31—H31B	109.5
C1—N1—C13	126.64 (13)	H31A—C31—H31B	109.5
C19—N2—C30	108.58 (12)	C15—C31—H31C	109.5
C19—N2—C17	126.05 (13)	H31A—C31—H31C	109.5
C30—N2—C17	125.37 (13)	H31B—C31—H31C	109.5
C43—N3—C32	108.49 (12)	C33—C32—N3	129.74 (14)
C43—N3—C44	125.60 (12)	C33—C32—C37	121.70 (14)
C32—N3—C44	125.82 (13)	N3—C32—C37	108.53 (13)
C61—N4—C50	108.59 (12)	C34—C33—C32	117.41 (15)
C61—N4—C46	125.26 (13)	C34—C33—H33A	121.3
C50—N4—C46	125.96 (13)	C32—C33—H33A	121.3
C2—C1—N1	129.75 (15)	C33—C34—C35	122.09 (16)
C2—C1—C6	121.52 (15)	C33—C34—H34A	119.0
N1—C1—C6	108.73 (14)	C35—C34—H34A	119.0
C1—C2—C3	117.75 (16)	C36—C35—C34	120.13 (15)
C1—C2—H2A	121.1	C36—C35—H35A	119.9
C3—C2—H2A	121.1	C34—C35—H35A	119.9
C2—C3—C4	121.60 (17)	C35—C36—C37	119.51 (16)
C2—C3—H3A	119.2	C35—C36—H36A	120.2
C4—C3—H3A	119.2	C37—C36—H36A	120.2
C5—C4—C3	120.33 (17)	C36—C37—C32	119.13 (15)
C5—C4—H4A	119.8	C36—C37—C38	133.62 (15)
C3—C4—H4A	119.8	C32—C37—C38	107.24 (13)
C4—C5—C6	119.59 (17)	C39—C38—C43	119.49 (15)
C4—C5—H5A	120.2	C39—C38—C37	133.61 (15)
C6—C5—H5A	120.2	C43—C38—C37	106.89 (14)
C5—C6—C1	119.20 (16)	C40—C39—C38	119.39 (16)
C5—C6—C7	133.86 (16)	C40—C39—H39A	120.3
C1—C6—C7	106.94 (14)	C38—C39—H39A	120.3
C8—C7—C12	119.39 (17)	C39—C40—C41	120.32 (16)
C8—C7—C6	133.62 (17)	C39—C40—H40A	119.8
C12—C7—C6	106.96 (14)	C41—C40—H40A	119.8
C9—C8—C7	118.77 (18)	C42—C41—C40	121.75 (16)
C9—C8—H8A	120.6	C42—C41—H41A	119.1
C7—C8—H8A	120.6	C40—C41—H41A	119.1
C8—C9—C10	121.21 (18)	C41—C42—C43	117.60 (15)
C8—C9—H9A	119.4	C41—C42—H42A	121.2
C10—C9—H9A	119.4	C43—C42—H42A	121.2
C11—C10—C9	121.41 (19)	C42—C43—N3	129.67 (14)
C11—C10—H10A	119.3	C42—C43—C38	121.41 (15)
C9—C10—H10A	119.3	N3—C43—C38	108.85 (13)
C10—C11—C12	117.48 (18)	C49—C44—C45	120.48 (14)
C10—C11—H11A	121.3	C49—C44—N3	119.91 (13)
C12—C11—H11A	121.3	C45—C44—N3	119.61 (14)
C11—C12—N1	129.33 (16)	C46—C45—C44	118.86 (14)
C11—C12—C7	121.71 (16)	C46—C45—H45A	120.6
N1—C12—C7	108.94 (15)	C44—C45—H45A	120.6
C14—C13—C18	120.26 (14)	C45—C46—C47	120.75 (14)
C14—C13—N1	119.67 (14)	C45—C46—N4	119.74 (14)
C18—C13—N1	120.04 (13)	C47—C46—N4	119.50 (14)
C15—C14—C13	120.68 (15)	C46—C47—C48	120.66 (14)
C15—C14—H14A	119.7	C46—C47—H47A	119.7
C13—C14—H14A	119.7	C48—C47—H47A	119.7
C14—C15—C16	119.13 (14)	C49—C48—C47	118.40 (14)
C14—C15—C31	120.20 (15)	C49—C48—C62	120.55 (14)
C16—C15—C31	120.65 (16)	C47—C48—C62	121.04 (14)
C15—C16—C17	120.12 (15)	C44—C49—C48	120.79 (14)
C15—C16—H16A	119.9	C44—C49—H49A	119.6
C17—C16—H16A	119.9	C48—C49—H49A	119.6
C18—C17—C16	120.46 (14)	C51—C50—N4	129.30 (14)
C18—C17—N2	119.41 (13)	C51—C50—C55	122.04 (14)
C16—C17—N2	120.12 (14)	N4—C50—C55	108.66 (13)
C17—C18—C13	119.33 (14)	C52—C51—C50	117.46 (15)
C17—C18—H18A	120.3	C52—C51—H51A	121.3
C13—C18—H18A	120.3	C50—C51—H51A	121.3
C20—C19—N2	129.55 (14)	C51—C52—C53	121.46 (17)
C20—C19—C24	121.80 (15)	C51—C52—H52A	119.3
N2—C19—C24	108.65 (14)	C53—C52—H52A	119.3
C21—C20—C19	117.52 (16)	C54—C53—C52	120.69 (16)
C21—C20—H20A	121.2	C54—C53—H53A	119.7
C19—C20—H20A	121.2	C52—C53—H53A	119.7
C20—C21—C22	121.40 (17)	C53—C54—C55	119.34 (15)
C20—C21—H21A	119.3	C53—C54—H54A	120.3
C22—C21—H21A	119.3	C55—C54—H54A	120.3
C23—C22—C21	121.01 (16)	C54—C55—C50	119.01 (15)
C23—C22—H22A	119.5	C54—C55—C56	134.08 (14)
C21—C22—H22A	119.5	C50—C55—C56	106.89 (13)
C22—C23—C24	118.94 (16)	C57—C56—C61	119.16 (15)
C22—C23—H23A	120.5	C57—C56—C55	133.81 (14)
C24—C23—H23A	120.5	C61—C56—C55	107.00 (13)
C23—C24—C19	119.31 (15)	C58—C57—C56	119.17 (15)
C23—C24—C25	133.59 (15)	C58—C57—H57A	120.4
C19—C24—C25	107.09 (13)	C56—C57—H57A	120.4
C26—C25—C30	119.45 (15)	C57—C58—C59	120.76 (15)
C26—C25—C24	133.71 (15)	C57—C58—H58A	119.6
C30—C25—C24	106.83 (13)	C59—C58—H58A	119.6
C27—C26—C25	119.14 (16)	C60—C59—C58	121.53 (16)
C27—C26—H26A	120.4	C60—C59—H59A	119.2
C25—C26—H26A	120.4	C58—C59—H59A	119.2
C26—C27—C28	120.67 (16)	C59—C60—C61	117.44 (14)
C26—C27—H27A	119.7	C59—C60—H60A	121.3
C28—C27—H27A	119.7	C61—C60—H60A	121.3
C29—C28—C27	121.84 (18)	C60—C61—N4	129.20 (13)
C29—C28—H28A	119.1	C60—C61—C56	121.93 (14)
C27—C28—H28A	119.1	N4—C61—C56	108.84 (14)
C28—C29—C30	117.28 (16)	C48—C62—H62A	109.5
C28—C29—H29A	121.4	C48—C62—H62B	109.5
C30—C29—H29A	121.4	H62A—C62—H62B	109.5
C29—C30—N2	129.49 (15)	C48—C62—H62C	109.5
C29—C30—C25	121.62 (15)	H62A—C62—H62C	109.5
N2—C30—C25	108.85 (14)	H62B—C62—H62C	109.5
			
C12—N1—C1—C2	−179.43 (16)	C43—N3—C32—C33	178.35 (16)
C13—N1—C1—C2	3.4 (3)	C44—N3—C32—C33	1.7 (3)
C12—N1—C1—C6	−0.39 (17)	C43—N3—C32—C37	0.38 (17)
C13—N1—C1—C6	−177.55 (14)	C44—N3—C32—C37	−176.22 (14)
N1—C1—C2—C3	177.99 (15)	N3—C32—C33—C34	−179.80 (16)
C6—C1—C2—C3	−1.0 (2)	C37—C32—C33—C34	−2.1 (2)
C1—C2—C3—C4	0.9 (2)	C32—C33—C34—C35	1.1 (3)
C2—C3—C4—C5	−0.4 (3)	C33—C34—C35—C36	0.5 (3)
C3—C4—C5—C6	−0.1 (2)	C34—C35—C36—C37	−1.1 (3)
C4—C5—C6—C1	0.1 (2)	C35—C36—C37—C32	0.2 (2)
C4—C5—C6—C7	−179.48 (17)	C35—C36—C37—C38	179.64 (17)
C2—C1—C6—C5	0.5 (2)	C33—C32—C37—C36	1.4 (2)
N1—C1—C6—C5	−178.65 (13)	N3—C32—C37—C36	179.59 (14)
C2—C1—C6—C7	−179.86 (14)	C33—C32—C37—C38	−178.14 (14)
N1—C1—C6—C7	1.00 (17)	N3—C32—C37—C38	0.03 (17)
C5—C6—C7—C8	−3.6 (3)	C36—C37—C38—C39	1.5 (3)
C1—C6—C7—C8	176.83 (17)	C32—C37—C38—C39	−179.01 (18)
C5—C6—C7—C12	178.35 (17)	C36—C37—C38—C43	−179.89 (17)
C1—C6—C7—C12	−1.23 (17)	C32—C37—C38—C43	−0.42 (17)
C12—C7—C8—C9	0.3 (2)	C43—C38—C39—C40	−1.8 (2)
C6—C7—C8—C9	−177.59 (17)	C37—C38—C39—C40	176.64 (17)
C7—C8—C9—C10	−1.6 (3)	C38—C39—C40—C41	0.0 (3)
C8—C9—C10—C11	1.8 (3)	C39—C40—C41—C42	1.4 (3)
C9—C10—C11—C12	−0.6 (3)	C40—C41—C42—C43	−0.9 (2)
C10—C11—C12—N1	177.13 (16)	C41—C42—C43—N3	−177.46 (15)
C10—C11—C12—C7	−0.7 (2)	C41—C42—C43—C38	−1.0 (2)
C1—N1—C12—C11	−178.50 (16)	C32—N3—C43—C42	176.13 (16)
C13—N1—C12—C11	−1.3 (3)	C44—N3—C43—C42	−7.3 (3)
C1—N1—C12—C7	−0.41 (17)	C32—N3—C43—C38	−0.65 (17)
C13—N1—C12—C7	176.81 (14)	C44—N3—C43—C38	175.96 (14)
C8—C7—C12—C11	0.9 (2)	C39—C38—C43—C42	2.4 (2)
C6—C7—C12—C11	179.28 (15)	C37—C38—C43—C42	−176.44 (14)
C8—C7—C12—N1	−177.37 (14)	C39—C38—C43—N3	179.48 (14)
C6—C7—C12—N1	1.02 (17)	C37—C38—C43—N3	0.65 (17)
C12—N1—C13—C14	58.8 (2)	C43—N3—C44—C49	134.06 (16)
C1—N1—C13—C14	−124.46 (17)	C32—N3—C44—C49	−49.9 (2)
C12—N1—C13—C18	−119.32 (17)	C43—N3—C44—C45	−46.4 (2)
C1—N1—C13—C18	57.4 (2)	C32—N3—C44—C45	129.62 (16)
C18—C13—C14—C15	−0.9 (3)	C49—C44—C45—C46	0.2 (2)
N1—C13—C14—C15	−179.08 (15)	N3—C44—C45—C46	−179.35 (14)
C13—C14—C15—C16	0.7 (3)	C44—C45—C46—C47	1.5 (2)
C13—C14—C15—C31	−177.85 (17)	C44—C45—C46—N4	−179.82 (14)
C14—C15—C16—C17	0.1 (3)	C61—N4—C46—C45	118.59 (17)
C31—C15—C16—C17	178.67 (17)	C50—N4—C46—C45	−67.0 (2)
C15—C16—C17—C18	−0.7 (2)	C61—N4—C46—C47	−62.7 (2)
C15—C16—C17—N2	178.26 (15)	C50—N4—C46—C47	111.75 (18)
C19—N2—C17—C18	123.85 (17)	C45—C46—C47—C48	−1.2 (2)
C30—N2—C17—C18	−56.5 (2)	N4—C46—C47—C48	−179.92 (14)
C19—N2—C17—C16	−55.1 (2)	C46—C47—C48—C49	−0.7 (2)
C30—N2—C17—C16	124.50 (17)	C46—C47—C48—C62	179.13 (16)
C16—C17—C18—C13	0.5 (2)	C45—C44—C49—C48	−2.1 (2)
N2—C17—C18—C13	−178.47 (14)	N3—C44—C49—C48	177.40 (14)
C14—C13—C18—C17	0.3 (2)	C47—C48—C49—C44	2.4 (2)
N1—C13—C18—C17	178.44 (14)	C62—C48—C49—C44	−177.48 (15)
C30—N2—C19—C20	178.56 (16)	C61—N4—C50—C51	−178.61 (16)
C17—N2—C19—C20	−1.8 (3)	C46—N4—C50—C51	6.2 (3)
C30—N2—C19—C24	−0.78 (17)	C61—N4—C50—C55	1.23 (17)
C17—N2—C19—C24	178.90 (14)	C46—N4—C50—C55	−173.99 (14)
N2—C19—C20—C21	−179.87 (16)	N4—C50—C51—C52	179.64 (16)
C24—C19—C20—C21	−0.6 (2)	C55—C50—C51—C52	−0.2 (2)
C19—C20—C21—C22	−0.6 (3)	C50—C51—C52—C53	0.9 (3)
C20—C21—C22—C23	0.9 (3)	C51—C52—C53—C54	−1.0 (3)
C21—C22—C23—C24	0.1 (3)	C52—C53—C54—C55	0.4 (3)
C22—C23—C24—C19	−1.3 (2)	C53—C54—C55—C50	0.2 (2)
C22—C23—C24—C25	179.13 (17)	C53—C54—C55—C56	−178.33 (17)
C20—C19—C24—C23	1.6 (2)	C51—C50—C55—C54	−0.4 (2)
N2—C19—C24—C23	−179.01 (14)	N4—C50—C55—C54	179.78 (14)
C20—C19—C24—C25	−178.74 (14)	C51—C50—C55—C56	178.56 (15)
N2—C19—C24—C25	0.66 (17)	N4—C50—C55—C56	−1.29 (17)
C23—C24—C25—C26	0.9 (3)	C54—C55—C56—C57	1.5 (3)
C19—C24—C25—C26	−178.68 (17)	C50—C55—C56—C57	−177.22 (17)
C23—C24—C25—C30	179.31 (17)	C54—C55—C56—C61	179.58 (17)
C19—C24—C25—C30	−0.30 (17)	C50—C55—C56—C61	0.88 (17)
C30—C25—C26—C27	−0.2 (2)	C61—C56—C57—C58	0.1 (2)
C24—C25—C26—C27	178.01 (17)	C55—C56—C57—C58	178.03 (16)
C25—C26—C27—C28	0.1 (3)	C56—C57—C58—C59	−0.5 (2)
C26—C27—C28—C29	−0.2 (3)	C57—C58—C59—C60	0.7 (3)
C27—C28—C29—C30	0.5 (3)	C58—C59—C60—C61	−0.5 (2)
C28—C29—C30—N2	−178.15 (17)	C59—C60—C61—N4	−177.70 (15)
C28—C29—C30—C25	−0.7 (3)	C59—C60—C61—C56	0.1 (2)
C19—N2—C30—C29	178.33 (17)	C50—N4—C61—C60	177.37 (16)
C17—N2—C30—C29	−1.4 (3)	C46—N4—C61—C60	−7.4 (3)
C19—N2—C30—C25	0.59 (17)	C50—N4—C61—C56	−0.66 (17)
C17—N2—C30—C25	−179.09 (14)	C46—N4—C61—C56	174.60 (14)
C26—C25—C30—C29	0.5 (2)	C57—C56—C61—C60	0.1 (2)
C24—C25—C30—C29	−178.12 (15)	C55—C56—C61—C60	−178.35 (14)
C26—C25—C30—N2	178.48 (14)	C57—C56—C61—N4	178.29 (14)
C24—C25—C30—N2	−0.18 (17)	C55—C56—C61—N4	−0.14 (17)

### Hydrogen-bond geometry (Å, º)

*Cg*1, *Cg*3—*Cg*6 and *Cg*9 are the centroids of the C44–C49, C7–C12, C56–C61, C1–C6, C19–C24 and C32–C37 benzene rings while *Cg*2, *Cg*7 and *Cg*8 are the centroids of the N4/C50/C55/C56/C61, N2/C19/C24/C25/C30 and N3/C32/C37/C38/C43 pyrrole rings.

**Table:**

*D*—H···*A*	*D*—H	H···*A*	*D*···*A*	*D*—H···*A*
C9—H9*A*···*Cg*1^i^	0.95	2.96	3.776 (2)	145
C5—H5*A*···*Cg*2^ii^	0.95	2.77	3.6969 (19)	165
C53—H53*A*···*Cg*3^iii^	0.95	2.76	3.5983 (19)	148
C8—H8*A*···*Cg*4^ii^	0.95	2.81	3.7122 (19)	160
C49—H49*A*···*Cg*5	0.95	2.98	3.8654 (16)	156
C57—H57*A*···*Cg*6^iv^	0.95	2.85	3.3776 (17)	117
C58—H58*A*···*Cg*7^iv^	0.95	2.88	3.3749 (18)	114
C59—H59*A*···*Cg*8^v^	0.95	2.66	3.5084 (18)	149
C60—H60*A*···*Cg*9^v^	0.95	2.74	3.5180 (17)	140

Symmetry codes: (i) *x*−1/2, −*y*+3/2, *z*+1/2; (ii) *x*+1/2, −*y*+3/2, *z*+1/2; (iii) *x*, *y*, *z*−1; (iv) −*x*, −*y*+1, −*z*+1; (v) *x*−1, *y*, *z*.

### Table 1. C—H···π-electron ring interactions. The centroids *Cg*1, *Cg*2, *Cg*3, *Cg*4, *Cg*5, *Cg*6, *Cg*7, *Cg*8 and *Cg*9 are the centroids of the respective rings C44–C49, N4/C50/C55/C56/C61, C7—C12, C56—C61, C1—C6, C19—C24, N2/C19/C24/C25/C30, N3/C32/C37/C38/C43 and C32—C37. The centroids *Cg*1, *Cg*3—*Cg*6 and *Cg*9 are pertinent to the benzene rings while the centroids *Cg*2, *Cg*7 and *Cg*8 are pertinent to the pyrrole rings.

**Table:**

C—H···*Cg*	D-H	H···*Cg*	D···*Cg*	D-H···*Cg*
C9-H9A··· *Cg*1^i^	0.95	2.959	3.776 (2)	145
C5-H5A··· *Cg*2^ii^	0.95	2.770	3.6969 (19)	165
C53-H53A··· *Cg*3^iii^	0.95	2.755	3.5983 (19)	148
C8-H8A··· *Cg*4^ii^	0.95	2.805	3.7122 (19)	160
C49-H49A··· *Cg*5	0.95	2.977	3.8654 (16)	156
C57-H57A··· *Cg*6^iv^	0.95	2.845	3.3776 (17)	117
C58-H58A··· *Cg*7^iv^	0.95	2.878	3.3749 (18)	114
C59-H59A··· *Cg*8^v^	0.95	2.658	3.5084 (18)	149
C60-H60A··· *Cg*9^v^	0.95	2.738	3.5180 (17)	140

Symmetry codes: (i) *x* - 1/2, -*y* + 3/2, *z* + 1/2; (ii) *x* + 1/2, -*y* + 3/2, *z* + 1/2; (iii) *x*, *y*, *z* - 1; (iv) -*x*, -*y* + 1, -*z* + 1; (v) *x* - 1, *y*, *z*.

### Table 2. Distances (Å) between the centroids of the rings involved in the π-electron ring···π-electron ring interactions which are present in the title structure. The centroids *Cg*10, *Cg*11 and *Cg*12 are pertinent to the benzene, pyrrole and benzene rings, respectively.

**Table:**

*Cg*i···*Cg*j	distance (Å)	1-^st^ ring	2-^nd^ ring
*Cg*10···*Cg*10^vi^	3.7698 (10)	C38//C39···C43	C38//C39···C43
*Cg*11···*Cg*11^vii^	3.8292 (9)	N2//C19···C30	N2//C19···C30
*Cg*12···*Cg*11^vi^	3.9429 (10)	C25//C26···C30	N2//C19···C30
*Cg*11···*Cg*12^vi^	3.9431 (10)	N2//C19···C30	C25//C26···C30

Symmetry codes: (vi) -*x* + 1, -*y* + 1, -*z* + 1; (vii) *x* + 3/2, -*y* + 3/2, *z* + 5/2.
